# Impact of the first COVID-19 lockdown on the relationship with parents and peers in a cohort of adolescents with somatic symptom disorder

**DOI:** 10.1186/s13052-022-01300-y

**Published:** 2022-06-20

**Authors:** Andrea Trombetta, Laura De Nardi, Giorgio Cozzi, Luca Ronfani, Lara Bigolin, Egidio Barbi, Matteo Bramuzzo, Giuseppe Abbracciavento

**Affiliations:** 1 Department of Medical, Surgical and Health Sciences, University of Trieste, Via dell’Istria 65/1, 34137 Trieste, Italy; 2 Institute for Maternal and Child Health IRCCS “Burlo Garofolo”, Via dell’Istria 65/1, 34147 Trieste, Italy

**Keywords:** COVID-19, Adolescent Health, Somatic symptom disorder, Mental health, Coping strategies

## Abstract

To investigate the coping strategies of a group of adolescents with somatic symptom disorder compared to non-somatic symptom disorder peers during the COVID-19 related lockdown.

This cross-sectional study is the second part of a previously published study showing an improved trend in depression and anxiety in a group of patients with somatic symptom disorder compared to non-somatic symptom disorder peers. An anonymous semi-structured survey was distributed to two groups of Italian adolescents to measure the impact of quarantine on their daily life and coping strategies.

We recruited 115 adolescents, 58 (50.4%) mean age 15.3, with a recent diagnosis of somatic symptom disorder and 57 (49.6%) mean age 15.8, control peers.

The aim of this study was to detect differences in coping strategies and relationships with parents and peers, during the lockdown period in a group of adolescents with somatic symptom disorder and low disease burden when compared with a non-somatic symptom disorder group.

The relationship with parents significantly worsened in 4 (6.9%) of adolescents with somatic symptom disorder compared to 12 (21.1%) adolescents in the non-somatic symptom disorder group (*p* = *0.048*). The relationship with peers significantly improved in 13 (22.4%) of adolescents with symptom disorder versus 3 (5.3%) of peers of the non- somatic symptom disorder group *(p* = *0.013)*.

Adolescents with somatic symptom disorder with a low burden of physical symptoms experienced less deterioration in their relationships with parents and peers than the non-somatic symptom disorder group.

**Supplementary Information:**

The online version contains supplementary material available at 10.1186/s13052-022-01300-y.

## Introduction

Italy is one of the countries most affected by the Coronavirus Disease 2019 (COVID-19) pandemic [1]. Due to the exponential spread of infected people and proportional deaths, the Italian government imposed a national lockdown from March 9 to May 4, 2020. As part of the lockdown process, all schools were closed, extracurricular and sports activities were suspended, and people movement from home was allowed only for emergencies. Therefore, children and adolescents were subjected to a strict ban on leaving their homes without justified reasons.

The reported mental health consequences of quarantine in the pediatric population consisted of a growing and widespread state of anxiety, fear, anger, and uncertainty [2]. Adolescents affected by already diagnosed psychological and neuropsychiatric disorders were at higher risk of flaring up their condition or developing new mental health disorders [3, 4].

According to the Diagnostic and Statistical Manual of Mental Disorders-5 (DSM-V), Somatic symptom disorder (SSD) is a diagnosis that describes a cluster of patients who have distressing somatic symptoms, abnormal thoughts, feelings, and behaviors leading to disruption and distress of daily functioning [5]. SSD has a 10% prevalence in adolescence, coexisting with anxiety and depression in about 30% of patients [6], affecting patients and families’ quality of life causing school absenteeism [7], separation from their peers, and repeated medical evaluations or hospital admissions [8].

An obligated quarantine can be considered as a forced and unusual experimental setting. Studying its impact and understanding the reactions of patients and parents can help identify protective or adverse factors that play a role in SSD. This may help in further recognizing the role of different triggers and dynamics in developing treating strategies or better defining possible risk factors.

In a previous study, we investigated the impact of the national lockdown on psychological symptom burden in a cohort of Italian adolescents with SSD, demonstrating an improvement in depression and anxiety during the forced isolation period by using the Children’s Depression Inventory Short Form (CDI-2-SF) and the Multidimensional Anxiety Scale for Children Self Report (MASC-2-SR) questionnaires, respectively [9]. In particular, the T-score physical symptom domain of the MASC-2-SR was significantly lower in SSD adolescents when compared to non-SSD peers.

This further study aimed to examine the attitudes, behaviors and coping strategies of the same cohort of adolescents with low burden of disease, including the relationship with peers and parents.

## Methods

This research was part of a cross-sectional observational study conducted in May 2020 at the tertiary level, university teaching, children’s hospital, Institute for Maternal and Child Health of Trieste, Italy. Eligible participants were adolescents aged between 13 and 18 years, experiencing the Covid-19 pandemic restrictive measures.

The inclusion criteria were: (1) adolescents who received a diagnosis of SSD at the Institute, within the previous one year, confirmed by a neuro-psychiatric specialist according to the DSM-V, (2) youths who previously accessed the Institute for an acute injury or an acute organic disease in the same period, matched for age and sex. The exclusion criteria were considered the inability to understand the Italian language, the presence of cognitive impairment, a chronic disease, a neuropsychiatric disorder, or any history of non-investigated chronic pain.

All the adolescents of the SSD groups were clinically evaluated jointly by a trained paediatrician and a pediatric psychiatrist.

Adolescents in the control group were consecutively enrolled if admitted for acute trauma (fractures) or illnesses (appendicitis, testicle torsion, pneumonia) without any referred previous history of chronic illness or psychological problem.

The SSD diagnosis was based on a clinical interview conducted by a child neuropsychiatrist and symptom ratings according to the DSM-V criteria [5]. The electronic medical records of all patients admitted between May 2019 and May 2020 were reviewed to collect information about age, gender, symptoms, symptoms complained at admission, diagnostic test, specialist consult, previous hospitalizations, and final diagnosis, including SSD, at discharge.

Based on the defined criteria, we identified two groups:

1. SSD group: adolescents with SSD diagnosis;
2. Control group: adolescents admitted to the hospital for an acute problem.

A letter explaining the purpose of the study was sent to parents of adolescents who met the inclusion/exclusion criteria. Written informed consent was signed before participation. An anonymous semi-structured SurveyMonkey questionnaire was offered (see online Supplementary file 1) to both adolescents’ groups. It included a general demographic survey, a modified version of the Health Questionnaire Physical Symptoms 15 (PHQ15), the MASC-2-SR and the CDI-2-SF questionnaires. Data regarding the MASC-2-SR and the CDI-2-SF have already been published in another study analyzing depression and anxiety tendencies [9].

The general demographic survey included 18 questions with binary, multiple choices, and the possibility of providing open answers. For each enrolled patient, the following demographic variables were collected: age, gender, information on coping strategies during adolescents’ free-time, including the following main domains: ‘purpose of internet usage,’ ‘pattern of social media, videogames and other leisure usages,’ ‘relationships with peers and parents.’ We also added two questions about general worries and any perceived positive aspect about the ongoing pandemic period to describe adolescents’ general coping strategies during the quarantine period. The PHQ15 questionnaire was administered with the intended goal of quantifying the somatic symptom burden perceived by this cohort of patients [10]. This questionnaire, which investigated somatic symptoms or symptom clusters that accounted for over 90% of the physical complaints reported in the outpatient setting [11], was initially composed of 15 items scoring 0–2 for each. In this study it was administrated with 14 items, excluding the one strictly related to the sexual sphere. According to previous studies with the PHQ-15 [12], missing values were replaced with the mean value of the remaining items if the number of absent items was less than 20%. If the number of missing items in the scale exceeded 20%, the sum score was not computed and counted as missing. The overall results over 14 items were then weighted to 15 items, to compare the result of this study to other previous studies and reference ranges (see Supplementary file 2). The total PHQ-15 score ranges from 0 to 30, and scores of ≥ 5, ≥ 10, ≥ 15 represent mild, moderate, and severe levels of somatization [10]. To assess the anxiety tendency we used the MASC 2-SR which quantifies adolescents’ anxiety across ten domains: separation panic (SP), generalized anxiety disease (GAD), humiliation and refusal (HR), performance fear (PF), social anxiety (SA:T), obsessive compulsive (OC), panic (P), tension and restlessness (TR), physical symptoms (PS:T) and harms avoidance (HA). It investigates 50 items with a score from 0 to 3 for each item. *T-scores* are categorized into six classifications: Very Elevated (*score* 70 +), Elevated (*score* 65–69), Quite Elevated (*score* 60–64), High Average (*score* 55–59), Average (*score* 40–54), and Low (*score* < 40) [13].

The primary study outcome was to detect differences in attitudes and behaviors during the COVID-19 lockdown, including coping strategies and relationships with parents and peers, between SSD group and their healthy peers. Ethics Committee and approval number: IRB, RC 10/20.

The sample size of 94 (47 for each group) was predetermined to carry out the study, assuming a between groups minimum clinically significant difference of 5 in the mean MASC 2-SR total score.

G* power software, two sided Wilcoxon-Mann Whitney test, 2 groups).

Categorical data were presented as number and percentage, continuous data as median and interquartile range (IQR). Differences between groups (i.e., male vs female or healthy vs SSD) were evaluated with the chi-squared test for categorical data and with the non-parametric Mann–Whitney test for continuous data. The use of nonparametric tests is justified by the non-normal distribution of data, evaluated both visually and with the Kolmogorov–Smirnov test. Differences with p-value < 0.05 were considered statistically significant. Analysis was performed using SPSS version 23 (IBM, New York, USA).

## Results

The survey was offered to 160 teenagers. Forty-five subjects (28.1%) refused (22 in the SSD group and 23 in the control group) and 115 were enrolled, 58 (50.4%) with SSD (mean age 15.3 years, 48.3% males), and 57 (49.6%) control peers (mean age 15.8 years, 54.4% males). All subjects had been home-confined for nine weeks when they completed the survey.

### Adolescent behaviors during quarantine

Data related to adolescents coping strategies, including the purpose of Internet usage, the pattern of social media, videogames and other leisure usage, relationships with peers and parents, general worries, and any perceived positive aspects about the pandemic, are shown in Table 1.

**Table 1:** General demographic survey, analyzing adolescents adaptive behaviors

	**Healthy**	**SSD**	***P* value**
**Do you have a smartphone and/or a laptop with internet connection?**			
Yes	57 (100%)	57(98.3%)	1
No	0	1 (1.7%)	
**For which purposes do you usually use internet?**			NA
Social Network	38 (66.7%)	32 (55.2%)	
Watching video on YouTube/ listening to music	13 (22.8%)	9 (15.5%)	
Reading newspapers online	1 (1.8%)	2 (3.4%)	
Playing videogames	4 (7%)	9 (15.5%)	
Online lessons/homework/school research	5 (8.8%)	10 (17.2%)	
Video calling	0	2 (3.4%)	
Watching tv series/Netflix	1 (1.8%)	0	
**Do you use Social network? If yes, which one do you use more?**			NA
Facebook	3 (5.3%)	4 (6.9%)	
Instagram	49 (86%)	44 (75.9%)	
Twitter	2 (3.5%)	1 (1.7%)	
Whatsapp	1 (1.8%)	4 (6.9%)	
Tik tok	3 (5.3%)	2 (3.4%)	
Snapchat	1 (1,8%)	2 (3.4%)	
Viber	0	1 (1.7%)	
I do not use social network	6 (10.5%)	7 (12.1%)	
**How many hours have you been spending on social network during the ongoing pandemic?**			0.844
0–1 h	13 (22.8%)	12 (20.7%)	
1–2 h	9 (15.8%)	13 (22.4%)	
2–3 h	15 (26.3%)	10 (17.2%)	
3–4 h	9 (15.8%)	8 (13.8%)	
4–5 h	6 (10.5%)	9 (15.5%)	
5–6 h	2 (3.5%)	2 (3.4%)	
6–7 h	3 (5.3%)	3 (5.2%)	
> 7 h	0	1 (1.7%)	
**How many hours have you been spending on Netflix during the ongoing pandemic?**			0.370
0–1 h	22 (38.6%)	21 (36.2%)	
1–2 h	16 (28.1%)	11 (19%)	
2–3 h	10 (17.5%)	5 (8.6%)	
3–4 h	1 (1.8%)	4 (6.9%)	
4–5 h	1 (1.8%)	4 (6.9%)	
5–6 h	1 (1.8%)	2 (3.4%)	
6–7 h	1 (1.8%)	1 (1.7%)	
> 7 h	0	1 (1.7%)	
I do not use Netflix	5 (8.8%)	9 (15.5%)	
**How many hours have you been spending watching tv during the ongoing pandemic?**			0.709
0–1 h	40 (70.2%)	34 (58.6%)	
1–2 h	6 (10.5%)	12 (20.7%)	
2–3 h	6 (10.5%)	8 (13.8%)	
3–4 h	2 (3.5%)	2 (3.4%)	
4–5 h	1 (1.8%)	1 (1.7%)	
5–6 h	1 (1.8%)	1 (1.7%)	
6–7 h	1 (1.8%)	0	
> 7 h	0	0	
**How many hours have you been spending watching video on YouTube during the ongoing pandemic?**			0.854
0–1 h	33 (57.9%)	31 (53.4%)	
1–2 h	11 (19.3%)	14 (24.1%)	
2–3 h	7 (12.3%)	5 (8.6%)	
3–4 h	3 (5.3%)	2 (3.4%)	
4–5 h	1 (1.8%)	2 (3.4%)	
5–6 h	0	0	
6–7 h	0	0	
> 7 h	0	0	
I do not watch video on YouTube	2 (3.5%)	4 (6.9%)	
**How many hours have you been spending on individual study and/or online videolessons?**			0.169
0–1 h	4 (7%)	3 (5.2%)	
1–2 h	3 (5.3%)	4 (6.9%)	
2–3 h	13 (22.8%)	6 (10.3%)	
3–4 h	16 (28.1%)	15 (25.9%)	
4–5 h	3 (5.3%)	8 (13.8%)	
5–6 h	6 (10.5%)	10 (17.2%)	
6–7 h	4 (7%)	9 (15.5%)	
> 7 h	8 (14%)	3 (5.2%)	
**How many hours have you been spending playing videogames/PlayStation?**			0.270
0–1 h	30 (52.6%)	24 (41.4%)	
1–2 h	5 (8.8%)	6 (10.3%)	
2–3 h	6 (10.5%)	7 (12.1%)	
3–4 h	4 (7%)	6 (10.3%)	
4–5 h	1 (1.8%)	2 (3.4%)	
5–6 h	4 (7%)	1 (1.7%)	
6–7 h	3 (5.3%)	1 (1.7%)	
> 7 h	1 (1.8%)	0	
I do not play videogames	3 (5.3%)	11 (19%)	
**Which type of videogames do you usually play?**			NA
Sport videogames	12 (21.1%)	11 (19%)	
Shooter/fighting games	12 (21.1%)	17 (29.3%)	
Tactical/strategy games	13 (22.8%)	11 (19%)	
Adventure games	1 (1.8%)	1 (1.7%)	
Virtual reality games	1 (1.8%)	2 (3.4%)	
Cars videogames	1 (1.8%)	0	
I do not play videogames	27 (47.4%)	26 (44.8%)	
**How many hours have you been spending on reading?**			0.509
0–1 h	42 (73.7%)	39 (67.2%)	
1–2 h	12 (21.1%)	10 (17.2%)	
2–3 h	3 (5.3%)	6 (10.3%)	
3–4 h	0	1 (1.7%)	
4–5 h	0	1 (1.7%)	
5–6 h	0	1 (1.7%)	
6–7 h	0	0	
> 7 h	0	0	
I do not read	0	0	
**Do you have been enjoying any activity other than the mentioned ones? If yes, can you specify?**			NA
Playing a musical instrument/singing	6 (10.5%)	5 (8.6%)	
Playing sport, cycling, walking	25 (43.9%)	33 (56.9%)	
Drawing, painting, writing, bricolage	5 (8.8%)	9 (15.5%)	
Cooking	4 (7%)	4 (6.9%)	
Gardening	8 (14%)	2 (3.4%)	
Playing with brothers/sisters/board games	4 (7%)	3 (5.2%)	
I do not enjoy any other activity	19 (33.3%)	12 (20.7%)	
How would you consider your relationships with peers/friends during the ongoing pandemic?*			0.013
Improved	3 (5.3%)	13 (22.4%)	
The same	37 (64.9%)	36 (62.1%)	
Worsened	17(29.8%)	9 (15.5%)	
How would you consider relationships with your parents during the ongoing pandemic?*			0.048
Improved	20 (35.1%)	18 (31%)	
The same	25 (43.9%)	36 (62.1%)	
Worsened	12(21.1%)	4 (6.9%)	
**Which are your main worries about the ongoing pandemic period?**			NA
The possibility that further pandemic spreading could occur affecting my parents, relatives and friends	29 (50.1%)	32 (55.2%)	
The possibility that the ongoing restrictive measures could negatively impact on my family’s economic condition	18 (31.6%)	17 (29.3%)	
The possibility that a prolonged quarantine could negatively conditioning my relationships with friends	10 (17.5%)	11 (19%)	
I am worried about the possibility of not coming back to school because I do not believe that online teaching is the same in terms of educational quality	2 (3.5%)	0	
The possibility that it will take too much time to come back to normality	0	2 (3.4%)	
The impact of this isolation period on my person (psychological/on my sporting career)	0	2 (3.4%)	
I do not have any worry	0	3 (5.2%)	
**Do you perceive any positive side of this ongoing pandemic period?**			NA
A possible reduction in terms of pollution	23 (40.4%)	35 (60.3%)	
The chance of having more free time	7 (12.3%)	8 (13.8%)	
Having time to think of my own priorities and reflecting about my feature	21 (36.8%)	16 (27.6%)	
It will make people think about problems of our society	0	1 (1.7%)	
Learning to appreciate little things	2 (3.5%)	0	
More time to spend with parents that are no more busy with work	1 (1.8%)	2 (3.4%)	
Minor stress due to less weekly activities	2 (3.5%)	1 (1.7%)	
More time to work on myself and my self-improvement	1 (1.8%)	0	
I do not see any positive aspect	1 (1.8%)	3 (5.2%)	

*NA* Not applicable

^*^ = *p* < 0.05

### Purpose of internet usage, the pattern of social media and video game and leisure usage

Only slight differences were seen between the two groups in terms of internet time utilization patterns for social purposes and videogames pattern usage. The SSD group reported to prefer videogames usage rather than social media or television usage, albeit in absence of statistical significance.

### Relationships with peers and parents

During the lockdown the relationship with parents was reported to be worsened by 21.1% of adolescents in the control group compared to 6.9% in the SSD group *(p 0.013).* In 31% of the control group and in 35.1% of the SSD group this relationship improved; it remained unchanged in 62.1% of the control group and in 43.9% of the SSD group. Relationships with peers improved by 22.4% in the SSD group compared to 5.3% in the control group *(p 0.048).*

### General worries and perceived positive aspects about the pandemic

When asked about the main worries about the pandemic, the two groups answered similarly. The primary reported concerns were ‘the possibility that further pandemic spreading could occur affecting parents, relatives, and friends,’(55.2% in the SSD group versus 50.1% of the control group) ‘the possibility that the ongoing restrictive measures could negatively impact on their family’s economic condition’(29.3% in the SSD group versus 31.6% of the control group) and ‘the fear that a prolonged quarantine could negatively condition their relationships with friends’ (19% in the SSD group versus 17.5% of the control group).

### Health questionnaire physical symptoms (PHQ15) scoring

PHQ15 scoring in the SSD are displayed in Table 2. Among fifty-eight patients with SSD, twenty-five (43.1%) reported no somatic symptoms (“Low” range, 0–4 score), while nineteen (32.8%) were categorized in the “Mild” range (5–9 score) and eleven (19%) were in the “Moderate” range. Three patients (5.2%), all in the female group, were classified in the “Severe” score.

**Table 2:** PHQ-15 scoring (weighted PHQ-15 score) in male and female SSD individuals. Results are reported when normalized from 14 items (for puberal girls) to 13 items (for prepuberal girls and boys) to 15 items

Weighted PHQ-15 scoring	F (n.30)	M (n.28)
Low (score 0–4)	7 (23.3%)	18 (64.3%)
Mild (score 5–9)	10 (33.3%)	9 (32.1%)
Moderate (score 10–14)	10 (33.3%)	1 (3.6%)
Severe (≥ 15)	3 (10%)	0 (0%)
*Mean scoring (SD)*	*8.4 (4.6)*	*3.9 (3.2)*

## Discussion

This observational study shows that, after nine weeks of COVID-19-related home isolation, when compared to a control group of peers, a broader proportion of adolescents with SSD were more likely to have their relationship with peers and parents improved during the quarantine. This experience is of particular interest when considering the vital link between peers and parents’ relationship and SSD development in the long term period [14]. In the context of pandemic restrictions, the lockdown may have acted as a social relief for most adolescents with SSD by reducing competition with peers and family expectancies, as demonstrated by the low burden of physical symptoms, the hallmark of this condition, as found by the PHQ-15 and MASC-2SR questionnaire. On the other side, social avoidance is strongly correlated with SSD [15], and the comfort reported by patients may have been related to fewer physical symptoms [16].

One may speculate that home confinement could be perceived as a substantial stressor for healthy people and, conversely, as a relief for SSD patients. Although SSD subjects could have experienced reduced external stressors, they may also, on the contrary, have benefited from improved relationships with peers and adults. In this context of school closure and limitation of social relationships, adults' expectations were reduced, performance pressure was limited, and peer competition could be less evident, while solidarity could be increased. This study shows a trend toward a better relationship between SSD adolescents and their parents during isolation compared to healthy peers, thus suggesting a possible protective parental role. However, parents’ s relationship could also take on regressive connotations such as the constant presence of the parent, the continuous attention on the adolescent, reduced only by interference such as school and work.

In this extreme pandemic contest, the condition that characterized the phases of somatic symptoms of a psychogenic nature of adolescents was therefore recreated: dedicated attention and avoidance of sources of stress.

On this topic, albeit in absence of statistical significance, a different Internet pattern of usage was detected in the two groups, being less switched and with less total average time spent to the social media utilization and more switched to the videogames utilization in the SSD group when compared to the non-SSD group. This pattern may in part explain the better self-esteem trend in SSD-adolescents [9], as less involved in comparison with peers, as detailed by the upward social comparison and reinforcing spirals hypotheses and the displacement hypothesis [17].

On the other hand, the presence of intense virtual activity, which facilitated relationships, could reproduce some dynamics of conflict and competition among peers, explaining the similar proportion of adolescent with stable relationship with peers between the two groups [18]. Finally, a different role of social media use could be postulated due to its relevant role in adolescents with chronic illness [19]_._ It can be assumed that children with SSD were more comfortable during isolation in the context of remote school lessons and more intense use of social media than peers with a different lifestyle, more focused on outdoor activities and face-to-face relationships, thus explaining the higher proportion of patients having improved their relationships with peers.

A further possible explanation for the well-being of the SSD group may also be related to limited access to medical care, which could increase anxiety and confusion in the absence of a correct diagnosis or through repeated visits with no valuable results [20]. The pandemic may have also shifted the focus from somatic symptoms to the viral infection threat, which is well known for having a minor impact on young people.

This study has some limitations. First, PHQ-15 was not determined before the pandemic began, so it was difficult to determine its effect on this condition burden and it was not administered to the non-SSD group since it was not conceived for healthy population. However, the first part of the study in the same cohort, aimed at determining depressive and anxiety tendencies comparing the two groups, showed a reduction in physical symptoms in adolescents with SSD compared to the control group [9]. In particular, according to the PS:T domain of the MASC 2-SR T-score, SSD patients also reported significantly less physical symptoms burden when compared to the adolescents belonging to the control group (*p* < *0.05*). The second limitation was that the study involved a limited number of patients and that the questionnaire has not yet been administered after the lockdown, even if we continue to follow up both groups to assess anxiety and depression tendencies. Finally, a questionnaire performed at a particular time in an adolescent’s history can be subjected to a selection and recall bias.

The study also had some points of strength. One was the accurate definition of the SSD diagnosis, always made jointly by a pediatric psychiatrist and a pediatrician in a group of children in regular follow-up. A further element was the quality of the PHQ-15 and MASC2-SR validated questionnaire, exploring different somatic domains.

The interpretation of these results must be cautious; the isolation lasted about nine weeks in Italy, and more extended periods can lead to different results. Furthermore, physicians will have to pay close attention to the reopening of schools, which could put a rebound pressure on these patients. In this perspective, we suggest that specifically tailored strategies should be prepared and developed to help the adolescent cope with the return to normal activities.

## Conclusions

Adolescents with SSD and low burden of physical symptoms during COVID-19 related isolation reported a better relationship with peers and parents when compared to the control group. A deeper understanding of the relevance of specific stress triggers offered by the unusual and highly specific pandemic contest can facilitate understanding the treatment of these patients.

## Supplementary Information

**Additional file 1: Supplementary Data 1.** Semi-structured questionnaire.

**Additional file 2: Supplementary file 2.** Modified PHQ-15 questionnaire.

## Data Availability

All data generated during this paper are included in this published article.

## Abbreviations

CDI-2-SF: Children’s Depression Inventory Short Form
COVID-19: Coronavirus Disease 2019
DSM-V: Diagnostic and Statistical Manual of Mental Disorders-5
GAD: Generalized anxiety disease
HA: Harms avoidance
HR: Humiliation and refusal
IQR: Interquartile range
MASC-2-SR: Multidimensional Anxiety Scale for Children Self Report
OC: Obsessive compulsive
P: Panic
PF: Performance fear
PHQ15: Health Questionnaire Physical Symptoms 15
PS:T: Physical symptoms and
SSD: Somatic symptom disorder
SA:T: Social anxiety
SP: Separation panic
TR: Tension and restlessness

## References

[1] Italy: WHO Coronavirus Disease (COVID-19) Dashboard With Vaccination Data | WHO Coronavirus (COVID-19) Dashboard With Vaccination Data. https://covid19.who.int/region/euro/country/it.

[2] Sprang G, Silman M (2013). Posttraumatic stress disorder in parents and youth after health-related disasters. Disaster Med Public Health Prep.

[3] Lee J (2020). Mental health effects of school closures during COVID-19. Lancet Child Adolesc Heal.

[4] Guido CA, Loffredo L, Zicari AM, et al. The Impact of the COVID-19 Epidemic During the Lockdown on Children With the Pediatric Acute-Onset Neuropsychiatric Syndrome (PANDAS/PANS): The Importance of Environmental Factors on Clinical Conditions. Front Neurol. 2021;12:702356. 10.3389/fneur.2021.702356 PMC8385147 34456853

[5] American Psychiatric Association. Diagnostic and Statistical Manual of Mental Disorders. (American Psychiatric Association, 2013). 10.1176/appi.books.9780890425596.

[6] Swain MS, Henschke N, Kamper SJ, et al. An international survey of pain in adolescents. BMC Public Health. 2014;14:447. 10.1186/1471-2458-14-447 PMC4046513 24885027

[7] Cozzi G, Barbi E (2020). Chronic school absenteeism as a diagnostic clue for paediatricians. J Paediatr Child Health.

[8] Cozzi G, Barbi E (2019). Facing somatic symptom disorder in the emergency department. J Paediatr Child Health.

[9] De Nardi L, Abbracciavento G, Cozzi G, et al. Adolescents with somatic symptom disorder experienced less anxiety and depression than healthy peers during the first COVID‐19 lockdown. Acta Paediatr. 2021. apa.15877. 10.1111/apa.15877. 10.1111/apa.15877 PMC8250840 33862667

[10] Kroenke K, Spitzer RL, Williams JBW (2002). The PHQ-15: validity of a new measure for evaluating the severity of somatic symptoms. Psychosom Med.

[11] Kroenke K, Spitzer RL, Williams JBW, Löwe B (2010). The patient health questionnaire somatic, anxiety, and depressive symptom scales: a systematic review. Gen Hosp Psychiatry.

[12] Kocalevent RD, Hinz A, Brähler E (2013). Standardization of a screening instrument (PHQ-15) for somatization syndromes in the general population. BMC Psychiatry.

[13] Fraccaro RL, Stelnicki AM, Nordstokke DW (2015). Test review: multidimensional anxiety scale for children by J. S. March. Can J School Psychol.

[14] Stickley A, Koyanagi A, Koposov R (2016). Loneliness and its association with psychological and somatic health problems among Czech, Russian and U. S. adolescents. BMC Psychiatry.

[15] Landstedt E, Hammarstrom A, Winefield H (2015). How well do parental and peer relationships in adolescence predict health in adulthood?. Scand J Public Health.

[16] Olsson I, Dahl AA (2012). Avoidant personality problems - Their association with somatic and mental health, lifestyle, and social network. Comm Based Study Compr Psychiatry.

[17] Boers E, Afzali MH, Newton N (2019). Association of screen time and depression in adolescence. JAMA Pediatr.

[18] Larson R, Lee M (1996). The capacity to be alone as a stress buffer. J Soc Psychol.

[19] De Nardi L, Trombetta A, Ghirardo S (2020). Adolescents with chronic disease and social media: a cross-sectional study. Arch Dis Child.

[20] Morabito G, Barbi E, Cozzi G (2020). The unaware physician's role in perpetuating somatic symptom disorder. JAMA Pediatr.

