# Supplementary material for: Impact of the first COVID-19 lockdown on the relationship with parents and peers in a cohort of adolescents with somatic symptom disorder
Source: Ital J Pediatr. 2022 Jun 20;48:104. doi: 10.1186/s13052-022-01300-y (PMC9207852; doi:10.1186/s13052-022-01300-y)
Supplement: Supplementary file 1 — Additional file 1: Supplementary Data 1. Semi-structured questionnaire. [file 13052_2022_1300_MOESM1_ESM.docx]

**Supplementary Data 1: semi-structured questionnaire**

**Effetti dell’isolamento sociale secondario alla pandemia da Covid-19 sulla salute psico-fisica degli adolescenti**

***Questionario demografico e sulle strategie di coping degli adolescenti***

1. Età

2. Genere M F

3. Possiedi uno smartphone/laptop con una connessione ad internet?  Si  No

4. Per cosa utilizzi maggiormente Internet?

 Social network

 Visualizzazione video su YouTube

 Lettura online di giornali

 Videogames

 Altro (specifica)

1. Utilizzi i social network? Se sì, quale utilizzi maggiormente?

 Facebook

 Instagram

 Twitter

 Altro (specifica………………………)

1. In questo periodo di isolamento, quante ore passi ogni giorno sui social network (Facebook, Twitter, altro)?

 0-1

 1-2

 2-3

 3-4

 4-5

 5-6

 6-7

 >7 (specifica…………………..)

1. In questo periodo di isolamento quante ore trascorri quotidianamente guardando Netflix?

 0-1

 1-2

 2-3

 3-4

 4-5

 5-6

 6-7

 >7 (specifica ……………….)

8. In questo periodo di isolamento quante ore trascorri quotidianamente guardando la televisione?

 0-1

 1-2

 2-3

 3-4

 4-5

 5-6

 6-7

 >7 (specifica …………….)

9. In questo periodo di isolamento quante ore trascorri quotidianamente guardando video su YouTube?

 0-1

 1-2

 2-3

 3-4

 4-5

 5-6

 6-7

 >7 (specifica……………)

10. Quante ore dedichi quotidianamente alle lezioni online e/o allo studio individuale?

 0-1

 1-2

 2-3

 3-4

 4-5

 5-6

 6-7

 >7 (specifica…………..)

11. Quante ore dedichi quotidianamente a giocare a playstation/videogames?

 0-1

 1-2

 2-3

 3-4

 4-5

 5-6

 6-7

 >7 (specifica…………..)

12. Che tipo di videogames utilizzi? Indica la categoria di quelli più utilizzati tra quelli indicati o specifica nella sezione “altro”.

 Videogames di sport

 Sparatutto/picchiaduro

 Videogiochi tattici/ di strategia

 Non utilizzo videogames

 Altro ………………………………

13. Quanto ore dedichi quotidianamente alla lettura?

 0-1

 1-2

 2-3

 3-4

 4-5

 5-6

 6-7

 >7 (specifica)

14. Svolgi altre attività oltre a quelle sopraelencate? Se sì, potresti specificare quale? (Es. suonare uno strumento, disegnare, giardinaggio etc..)

 Suonare uno strumento

 Disegnare

 Non svolgo nessuna altra attività

 Altro……………………………

15. Come valuti i rapporti con i tuoi coetanei in questo periodo rispetto ai mesi scorsi precedenti l’isolamento? (Specifica perché)

 Migliorati (perché …………………………………………………………..)

 Uguali a prima (perché ……………………………………………………….)

 Peggiorati (perché ……………………………………………………………)

16. Come valuti i rapporti con i tuoi genitori in questo periodo rispetto ai mesi scorsi, precedenti l’isolamento? (Specifica perché)

 Migliorati (perché …………………………………………………………..)

 Uguali a prima (perché ………………………………………………………)

 Peggiorati (perché ……………………………………………………………)

17. Cosa ti preoccupa di più riguardo a quello che sta accadendo? Se sono presenti più condizioni elencate o altre cose, indicale nella sezione “altro”:

 La possibilità che la pandemia sia diffonda e colpisca i miei amici ed i miei familiari

 La possibilità che l’isolamento condizioni negativamente le possibilità lavorative e la condizione economica mia e dei miei familiari

 La possibilità che una quarantena prolungata condizioni i miei rapporti sociali, facendomi perdere amicizie

Altro ……………………………………………………………………

18. Ritieni che questo periodo di isolamento abbia un risvolto positivo? Se sì, quale? Se sono presenti più condizioni elencate o altre cose, indicacele nella sezione “altro”:

 Minore inquinamento

 Avere più tempo libero

 Possibilità di ripensare alle proprie priorità e riflettere sul proprio futuro

 Altro ………………………………………………………………………
