# Supplementary material for: Impact of the first COVID-19 lockdown on the relationship with parents and peers in a cohort of adolescents with somatic symptom disorder
Source: Ital J Pediatr. 2022 Jun 20;48:104. doi: 10.1186/s13052-022-01300-y (PMC9207852; doi:10.1186/s13052-022-01300-y)

During the past 4 weeks, how much have you been bothered by any of the following problems?

|  | Not bothered at all (0) | Bothered a little (1) | Bothered a lot (2) |
| --- | --- | --- | --- |
| Stomach pain |  |  |  |
| Back pain |  |  |  |
| Pain in your arms, legs, or joints (knees, hips, etc.) |  |  |  |
| Menstrual cramps or other problems with your periods WOMEN ONLY |  |  |  |
| Headaches |  |  |  |
| Chest pain |  |  |  |
| Dizziness |  |  |  |
| Fainting spells |  |  |  |
| Feeling your heart pound or race |  |  |  |
| Shortness of breath |  |  |  |
| Constipation, loose bowels, or diarrhea |  |  |  |
| Nausea, gas, or indigestion |  |  |  |
| Feeling tired or having low energy |  |  |  |
| Trouble sleeping |  |  |  |

(For office coding: Total Score T (x)_____ = _____ + _____ ). In females y= x (15/14). In males y= x (14/13)

**Supplemenatary file 2.** Modified PHQ-15 questionnaire


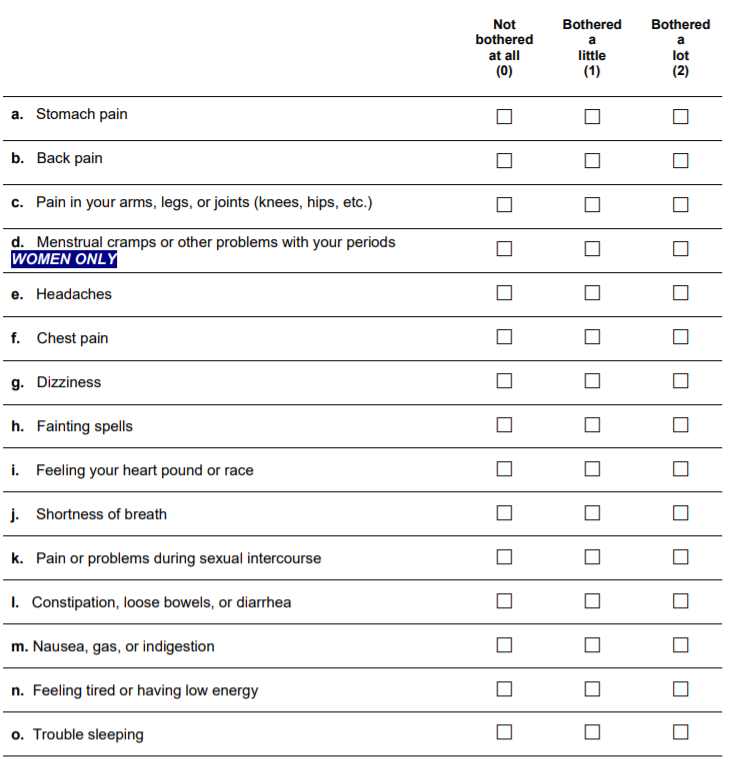

Supplement: Supplementary file 2 — Additional file 2: Supplementary file 2. Modified PHQ-15 questionnaire. [file 13052_2022_1300_MOESM2_ESM.docx]
